# Targeting hypoxia in tumor: a new promising therapeutic strategy

**DOI:** 10.1186/s13046-019-1517-0

**Published:** 2020-01-10

**Authors:** Maria Carla Bosco, Gabriella D’Orazi, Donatella Del Bufalo

**Affiliations:** 1 Laboratory of Molecular Biology, IRCCS Istituto Giannina Gaslini, 16147 Genova, Italy; 2 Department of Medical Science, University “G. D’Annunzio”, 66013 Chieti, Italy; 3 Unit of Cellular Networks and Molecular Therapeutic Targets, Department of Research and Advanced Technologies, IRCCS Regina Elena National Cancer Institute, 00144 Rome, Italy; 4 Preclinical Models and New Therapeutic Agents Unit, Department of Research and Advanced Technologies, IRCCS Regina Elena National Cancer Institute, 00144 Rome, Italy

**Keywords:** Hypoxia, HIF, Oxygen sensing, VHL, Angiogenesis, VEGF, Cancer, Metastasis

## Abstract

Low oxygen condition (hypoxia) is considered a hallmark of rapidly growing solid tumors. The presence of hypoxia renders tumor cells resistant to conventional chemo- and radio-therapy selecting a more malignant and invasive phenotype, and playing a negative role in patient prognosis. This commentary wishes to recognize the 2019 Nobel Prize in Medicine awarded to three physicians-scientists, Prof. William G. Kaelin Jr., Prof. Sir Peter J. Ratcliffe, and Prof. Gregg L. Semenza, for their discovery of the mechanisms mediating cell ability to sense and adapt to changes in oxygen availability. Their studies established the basis for our understanding of the role of hypoxia in a variety of diseases, including anemia, renal failure, cardiovascular disease, metabolic diseases, and cancer, paving the way for new promising therapeutic strategies through the development of drugs that can either activate or block the oxygen-sensing machinery.

## Background

**Fig. 1:**
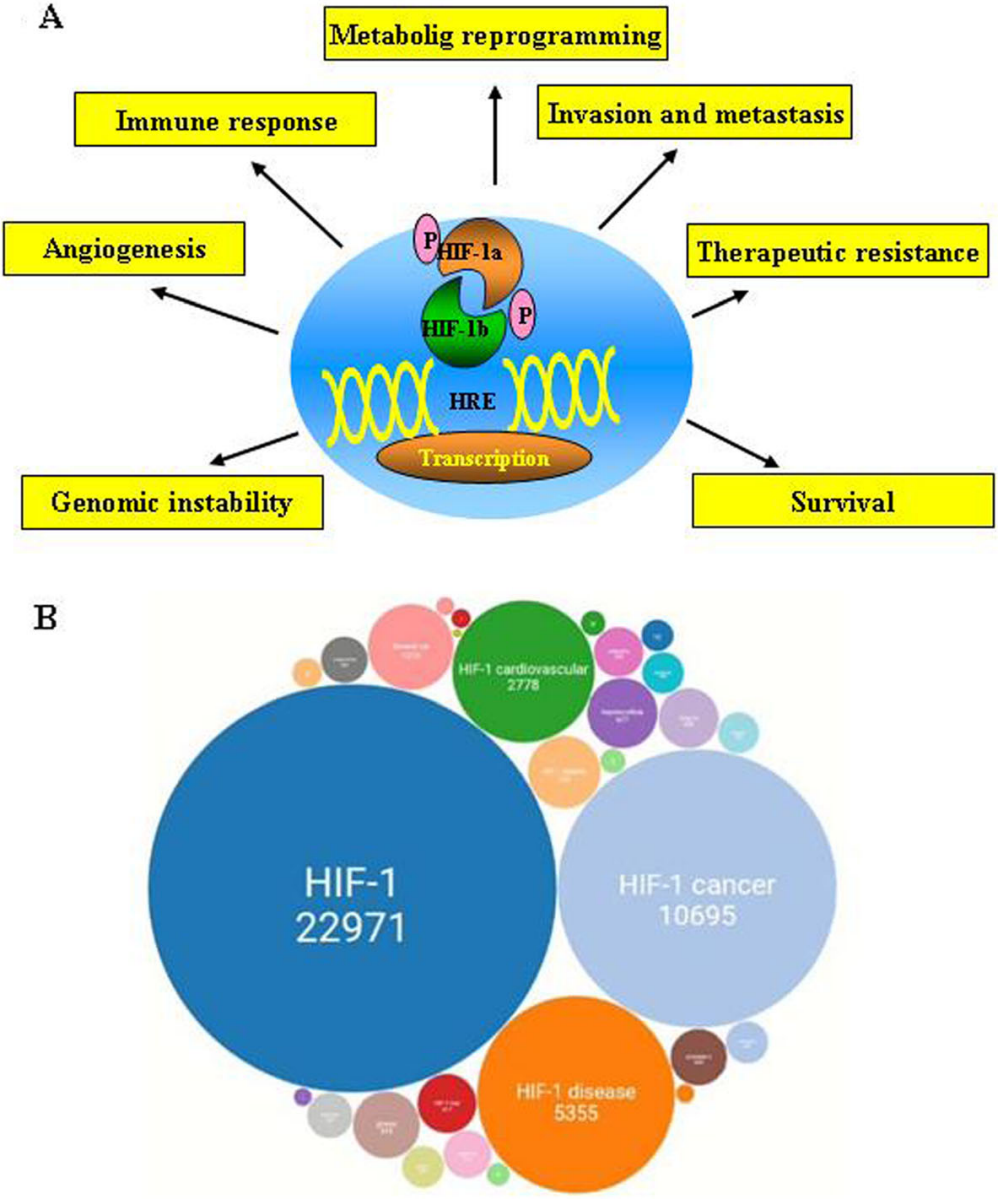
Schematic representation of the effects of intratumoral hypoxia in human diseases. **a** Hypoxia-inducible factor 1 (HIF-1) is a heterodimeric protein that consists of a constitutively expressed HIF-1β subunit and a HIF-1α subunit regulated via O_2_-independent mechanisms. Activated HIF-1 transcription factor binds the hypoxia response elements (HRE) to induce transcription of target genes involved in angiogenesis, glucose metabolism, cell proliferation/survival and invasion/metastasis, etc., as described in the panels. **b** Schematic representation of the number of scientific papers about HIF-1, published in the last 15 years, and the relative diseases

The fundamental importance of adequate oxygenation for energy production has been recognized for centuries, but how cells and tissues are able to sense and adapt to changes in oxygen availability remained elusive until the late twentieth century. In the last 30 years, three physician-scientists, William G. Kaelin Jr., Sir Peter J. Ratcliffe, and Gregg L. Semenza, focused their studies on the molecular mechanisms of oxygen detection in human and animal cells and the signaling pathways and biological processes by which they respond to low oxygen level, referred to as hypoxia. Their studies provided a series of closely overlapping and sometimes competitive contributions that ended with the Nobel Prize in Medicine 2019. Hypoxia Inducible Factor-1 (HIF-1), is an α/β heterodimeric transcription factor that controls multiple oxygen-sensitive genes. In 1995 Semenza identified HIF-1α as a basic-helix-loop-helix-PAS heterodimer regulated by cellular oxygen tension [1]. Then the three scientists independently identified different molecular mechanisms through which cells regulate HIF-1 activity and the downstream induced genes, among which, erythropoietin (EPO) [1] and angiogenic factors [2], modulating about 5% of the human genome (Fig. 1a) and affecting many different diseases, as evidenced by the increased number of papers published on this topic in the last 15 years (Fig. 1b). HIF-1-dependent induction of hypoxia-responsive genes has been shown in hypoxic regions of tumor xenografts, suggesting a connection between HIF and cancer. Ratcliffe and Kaelin demonstrated the relevance of the von Hippel–Lindau (VHL) tumor suppressor protein for HIF-1α degradation through oxygen-regulated prolyl hydroxylation [3, 4], and Semenza identified the factor inhibiting HIF-1 (FIH-1) as a novel protein interacting with HIF-1α and VHL gene to mediate repression of HIF-1 transcriptional activity [5]. Prolyl hydroxylation at specific sites of HIF-1α is the crucial oxygen-dependent post-translational modification required for its recognition and degradation by the VHL ubiquitin proteasome pathway. This process is blocked under hypoxic conditions because oxygen-requiring HIF hydroxylases no longer act and HIF-1α can be stabilized and assemble with HIF-1β dimerization partner, binding to the hypoxia response elements (HRE) of hypoxia-regulated genes and transactivating their expression [4] (Fig. 1a). Furthermore, regulation of HIF-1 activity has been shown to depend also by hypoxia-independent genetic alteration [3–5]. Starting from these studies, a plethora of regulators of HIF-1 transcriptional activity and HIF target genes affecting cellular responses to oxygen availability and, in particular, to hypoxic conditions, have been identified by researchers from all over the world [6, 7]. In 2010 the three scientists shared the Canada Gairdner International Award and in 2016 they received the Albert Lasker Award for Basic Medical Research for their work on oxygen sensing.

HIF-1 plays important roles in critical aspects of cancer biology, including angiogenesis, regulation of glucose and energy metabolism, epithelial-to-mesenchymal transition, invasion, and metastasis, and stem cell maintenance, thus allowing tumor cells to proliferate and survive under hypoxic conditions. These discoveries provided proof-of-principle that inhibition of HIF-1 activity may represent a novel strategy for the therapy not only of cancer but also for other diseases characterized by impaired oxygenation such as anemia, coronary artery disease, obstructive pulmonary diseases, chronic ischemic cardiomyopathy, inflammatory synovitis, atherosclerosis, systemic sclerosis, etc. [8, 9].

Over the last two decades, to bridge basic science to the clinical situation, dozens of putative small molecule HIF inhibitors that directly or indirectly downregulate HIF-1α have been identified and are currently tested in clinical trials for various forms of cancer [10]. Several HIF prolyl hydroxylase inhibitors, that prevent VHL from binding to HIF-1α, have also been developed and are now in late-stage clinical trials in disease in which HIF signaling is beneficial, e.g. to augment endogenous EPO production for the treatment of renal-based anemia. Such drugs are also being investigated for the treatment of circulatory diseases and for the protection against ischemic injury, inflammatory diseases, other than as anticancer molecules.

## Conclusion

Hypoxia is considered a driving force of tumor progression and a negative prognostic factor. The finding of HIF as the main regulator of transcriptional responses to changes in oxygen levels have far-reaching implications, opening up new avenues for the development of new promising therapeutic strategies targeting the HIF-signaling pathway. In this regard, *Journal of Experimental & Clinical Cancer Research* is announcing a special issue to highlight significant advances on the understanding of the impact of hypoxia on tumor progression and treatment efficacy.

## Data Availability

Not applicable.

## Abbreviations

EPO: Erythropoietin
FIH-1: Factor inhibiting HIF-1
HIF-1: Hypoxia Inducible Factor-1
HRE: Hypoxia response elements
VHL: Von Hippel–Lindau
