# Correction to: Targeting hypoxia in tumor: a new promising therapeutic strategy

**DOI:** 10.1186/s13046-020-1532-1

**Published:** 2020-02-24

**Authors:** Maria Carla Bosco, Gabriella D’. Orazi, Donatella Del Bufalo

**Affiliations:** 1 Laboratory of Molecular Biology, IRCCS Istituto Giannina Gaslini, 16147 Genoa, Italy; 2 Department of Medical Science, University “G. D’Annunzio”, 66013 Chieti, Italy; 3 Unit of Cellular Networks and Molecular Therapeutic Targets, Department of Research and Advanced Technologies, IRCCS Regina Elena National Cancer Institute, 00144 Rome, Italy; 4 Preclinical Models and New Therapeutic Agents Unit, Department of Research and Advanced Technologies, IRCCS Regina Elena National Cancer Institute, 00144 Rome, Italy

**Correction to: J Exp Clin Cancer Res (2020) 39:8.**

**https://doi.org/10.1186/s13046-019-1517-0**

In the original publication of this manuscript [1], Fig. 1 contained a typographical error (‘Metabolic’ incorrectly written as ‘Metabolig’). The caption for Fig. 1 also contained typographical errors; “… regulated via O2-independent mechanisms.” should be instead have read “… regulated by O_2_-dependent mechanisms.”, and the caption should refer to 25 years and not 15 years. Figure 1 and its caption have now been corrected and are shown below.

**Fig. 1:**
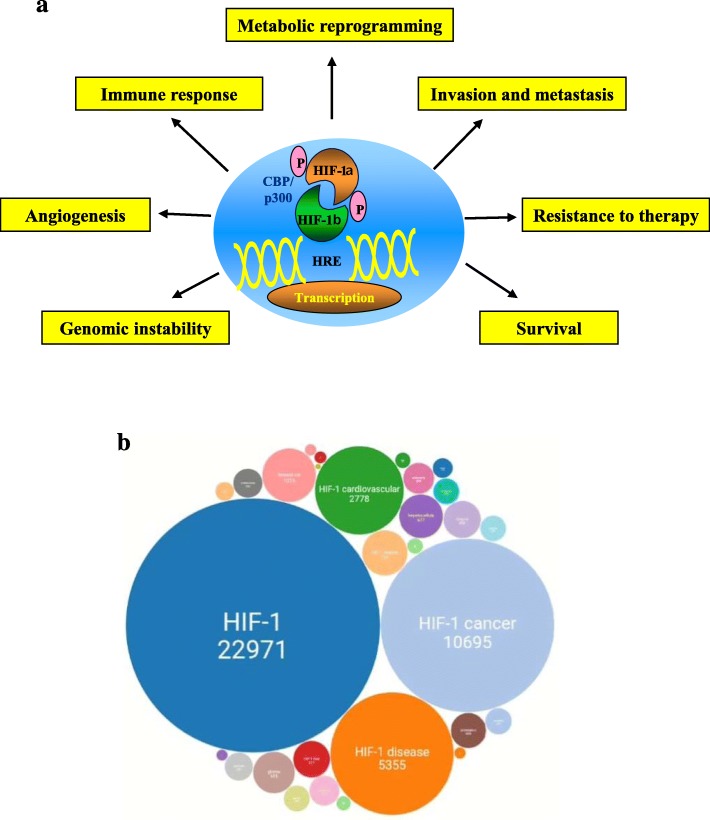
Schematic representation of the effects of intratumoral hypoxia in human diseases. **a** Hypoxia-inducible factor 1 (HIF-1) is a heterodimeric protein that consists of a constitutively expressed HIF-1β subunit and a HIF-1α subunit regulated by O_2_-dependent mechanisms. Activated HIF-1 transcription factor binds to the hypoxia response elements (HRE) to induce transcription of, among others, target genes involved in angiogenesis, glucose metabolism, cell proliferation/survival, and invasion/metastasis. **b** Schematic representation of the number of scientific papers related to HIF-1, published in the last 25 years, and the relative diseases

In addition, the following sentences have been adjusted to remove ambiguity and correct the record:

‘Background’ section, “Hypoxia Inducible Factor-1 (HIF-1), is an α/β heterodimeric transcription factor that controls multiple oxygen-sensitive genes. In 1995 Semenza identified HIF-1α as a basic-helix-loop-helix-PAS heterodimer regulated by cellular oxygen tension” has been corrected to “In 1995, Semenza identified the Hypoxia Inducible Factor-1 (HIF-1) as a basic-helix-loop-helix-PAS α/β heterodimeric transcription factor regulated by cellular oxygen tension.”

‘Background’ section, “… as evidenced by the increased number of papers published on this topic in the last 15 years” has been corrected to “… as evidenced by the increased number of papers published on this topic in the last 25 years.”

The authors sincerely apologize for the inconvenience caused to the readers. The original article has been updated.
